# A Multiobjective Hybrid Optimization Algorithm for Path Planning of Coal Mine Patrol Robot

**DOI:** 10.1155/2022/9094572

**Published:** 2022-06-23

**Authors:** Yongxin Gao, Zhonglin Dai, Jing Yuan

**Affiliations:** School of Mechanical Engineering, Liaoning Technical University, Fuxin 123000, China

## Abstract

In the complex underground environment, the paths planned for coal mine patrol robot are often too long and unsmooth under the influence of low visibility and poor road conditions. To solve the problems, this paper improves the hybrid algorithm between the improved artificial fish swarm algorithm (AFSA) and the dynamic window algorithm (DWA) for global path planning of coal mine patrol robot and introduces the improved genetic algorithm (GA) to enhance the path planning accuracy. Based on the global optimal path, the improved DWA was adopted to design a new adaptive trajectory evaluation function, which improves the ability of the patrol robot to avoid local obstacles. The proposed optimization algorithm was proved feasible through simulations. In addition, a simulation platform for the control of coal mine patrol robot was established, using the software development platform for coal mine patrol robot and robot operating system (ROS). The simulation results show that the improvement shortened the path length by 0.12 m, reduced the time by 3.14 s, and removed many turning points and redundant points. Therefore, the proposed improved hybrid path planning algorithm is effective and superior.

## 1. Introduction

The path planning of coal mine patrol robot, the key to autonomous navigation, falls into local path planning and global path planning [1–3]. It is important for coal mine patrol robot to search for the optimal path in the complex underground environment. The path optimization would significantly facilitate all underground operations and enhance the working efficiency of the robot.

Bayat et al. [4] improved the fixed step size and visual range in the traditional artificial fish swarm algorithm (AFSA) and proposed the visual range and step size based on the logarithmic function, thereby optimizing the path planning. According to the no free lunch (NFL) theorem, Yu et al. [5] put forward an intelligent bionic algorithm and utilized the algorithm collaboratively with other intelligent algorithms. The collaborative approach can plan better paths and apply them to a wider range than a single bionic path planning algorithm. Zhang et al. [6] improved the AFSA to improve the global search ability of robot. The improved AFSA (IAFSA) boasts a good real-time performance, achieves a high stability, and generates desirable paths at an acceptable time cost. Guo et al. [7] proposed an improved artificial fish swarm algorithm and combined the MS algorithm with the proposed IAFSA algorithm. The MS algorithm first calculates the top K shortest paths, and then the IAFSA algorithm quickly optimizes these paths. Zhou and Liu [8] proposed to adjust the viewable domain, movement step, and crowding factor of artificial fish by adaptive adjustment factor, so that the traversal of the algorithm can be improved to obtain the global optimum and achieve local search at the same time. Ma et al. [9] designed a UAV autonomous route planning method based on an ant colony algorithm that improves the optimization of artificial fish populations, which is faster and of higher quality than traditional methods. Zhang and Yu [10] integrated the optimization factors of the Tianniu whisker algorithm in the artificial fish swarm algorithm process, and the paths planned by the hybrid algorithm decreased significantly in terms of length and number of path vertices. Chang et al. [11] propose a better azimuth range constraint; that is, after simulating the pretrajectory, the better azimuth range is calculated according to the calculation criterion, then the pretrajectory within the better azimuth range at the next moment is selected, and finally the velocity corresponding to the optimal trajectory is determined by the trajectory evaluation function. Fox et al. [12] created the dynamic window algorithm (DWA). After determining the dynamic window, the DWA collects samples from the feasible velocity space, simulates each velocity sample to obtain the motion trajectory, and identifies the best velocity sample by the trajectory evaluation function. Based on the best sample, the robot is controlled to complete local path planning. To avoid dynamic obstacles, Chen [13] developed a DWA suitable for complex environments. The evaluation function was optimized to consider the distance increment of the robot moving towards the target point, and the minimum distance between the robot and the obstacle. The superiority of their DWA was demonstrated through simulation. The path planning method mentioned above has the disadvantages of poor search and development balance, slow convergence at the later stage of search, and inability to find the exact value. It is easy to fall into local optimum and convergence accuracy is not high.

In the complex underground environment, the paths planned for coal mine patrol robot are often too long and unsmooth. To solve the problems, this paper improves the hybrid AFSA-DWA path planning algorithm. The improved AFSA was adopted for global path planning of coal mine patrol robot, and the improved genetic algorithm (GA) was introduced to enhance the path planning accuracy of coal mine patrol robot. Based on the global optimal path, the improved DWA was adopted to design a new adaptive trajectory evaluation function, which improves the ability of the patrol robot to avoid local obstacles. It can make the planning path of coal mine inspection robot smoother, significantly reduce the small angle turning points and redundant points, and shorten the planning path length of coal mine inspection robot. Simulation results show that the hybrid algorithm enables coal mine patrol robot to plan paths more effectively and demonstrate the feasibility and effectiveness of our algorithm.

## 2. Path Planning Model

### 2.1. Mathematical Model

**Figure 1:**
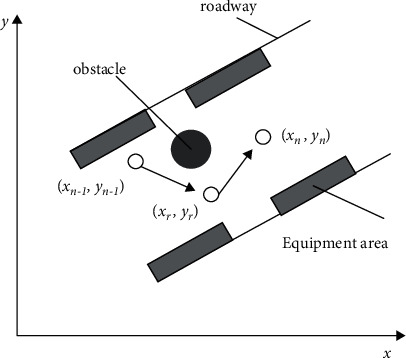
Path planning for coal mine patrol robot.

For a moving coal mine patrol robot, the shortest distance from the current point to the target point can be solved according to the path length [14]. Suppose there are *n* nodes on a path. Then, the path contains *n* − 1 segments. Each segment can be expressed as *l* = (*l*1, *l*2,…, *ln*), where li is a node on that path.  Figure 1 shows the robot path with the least redundancy and no chance of collision from the *n* − 1-th node (*x*_*n*_ − 1, *y*_*n*_ − 1) to the *n*-th node (*x*_*n*_, *y*_*n*_). When the coal mine inspection robot inspects the roadway, it needs to move over the obstacle to the next point, and the transition point (*x*_*r*_, *y*_*r*_) needs to be added between the two points affected by the obstacle so that the inspection robot can complete the inspection task.

As shown in Figure 1, the mathematical model for coal mine patrol robot to move from the *n* − 1-th node (*x*_*n*_ − 1, *y*_*n*_ − 1) to the *n*-th node (*x*_*n*_, *y*_*n*_) can be expressed as

(1) $$\begin{array}{c} l=\sqrt{{\left(x_{n-1}-x_{r}\right)}^{2}+{\left(y_{n-1}-y_{r}\right)}^{2}}+\sqrt{{\left(x_{r}-x_{n}\right)}^{2}+{\left(y_{r}-y_{n}\right)}^{2}}. \end{array}$$

Let *θ*_*n*_ − 1 and *V*_*n*_ − 1 be the turning angle and velocity of coal mine patrol robot at the *n* − 1-th node relative to the *X*-axis, respectively. Then, we have

(2) $$\begin{array}{c} \left\{\begin{array}{c} x_{r}=x_{n-1}+V_{n-1}\cos\;\theta_{n-1}\\ y_{r}=y_{n-1}+V_{n-1}\sin\;\theta_{n-1} \end{array}.\right. \end{array}$$

From formulas (1) and (2), the path from one point to another can be divided into the path planning from the starting point to the transition point and finally to the target point during the robot operation; based on this, the path planning model for coal mine patrol robot can be obtained as

(3) $$\begin{array}{c} l=V_{n-1}+\sqrt{{\left(x_{n-1}+V_{n-1}\cos\;\theta_{n-1}-x_{n}\right)}^{2}+{\left(y_{n-1}+V_{n-1}\sin\;\theta_{n-1}-y_{n}\right)}^{2}}, \end{array}$$

where *l* is the distance between two nodes, *V*_*n*_ − 1 is the velocity of robot at the *n* − 1-th node, and *θ*_*n*_ − 1 is the turning angle of robot at the *n*-th node.

### 2.2. Objective Function

The path planning of coal mine patrol robot mainly considers three optimization parameters: total path length, smoothness, and security.

#### 2.2.1. Total Path Length

When path *l* is feasible, the path length *f*_1_(*l*) can be expressed as

(4) $$\begin{array}{c} f_{1}\left(l\right)=\sum_{i=1}^{n}\left|l_{i}l_{i+1}\right|. \end{array}$$

When path *l* is infeasible, the path length *f*_1_(*l*) can be expressed as

(5) $$\begin{array}{c} f_{1}\left(l\right)=\sum_{i=1}^{n}\left|l_{i}l_{i+1}\right|+C, \end{array}$$

where |*l*_*i*_*l*_*i*+1_| is the length of segment *l*_*i*_*l*_*i*+1_ and *C* is the penalty term against infeasible path.

#### 2.2.2. Smoothness

For a path containing *n* nodes, there exist *n* − 2 angles between the segments. When path *l* is feasible, the smoothness *f*_2_(*θ*) of the path can be expressed as

(6) $$\begin{array}{c} f_{2}\left(l\right)=\frac{\left(\sum_{i=1}^{n-2}\theta_{i}\right)}{\left(n-2\right)}. \end{array}$$

When path *l* is infeasible, the smoothness *f*_2_(*θ*) of the path can be expressed as

(7) $$\begin{array}{c} f_{2}\left(l\right)=\frac{\left(\sum_{i=1}^{n-2}\theta_{i}\right)}{\left(n-2\right)}+C_{1}, \end{array}$$

where *θ*_*n*_ − 1 is the angle between segments li − 1li and lili + 1 and *C*1 is the penalty term against infeasible path.

#### 2.2.3. Security

When path *l* is feasible, the security *f*_3_(*l*) of the path can be expressed as

(8) $$\begin{array}{c} f_{3}\left(l\right)=\frac{1}{\left(g+a\right)}, \end{array}$$

where *g* is the shortest distance from the coal mine patrol robot to the obstacle on the planned path and a is the path security coefficient of coal mine patrol robot.

When path *l* is infeasible, the security *f*_3_(*l*) of the path can be expressed as

(9) $$\begin{array}{c} f_{3}\left(l\right)=C_{2}, \end{array}$$

where C2 is the penalty term against infeasible path.

Through the above analysis, proper weights a1, a2, and a3 were assigned to different optimization parameters, according to the importance of f1(l), f2(l), and f3(l) to the path planning of coal mine patrol robot. The three resulting weight matrices of the three objectives were multiplied with the matrix composed of the three optimization parameters, producing the objective function f(l) for path l:

(10) $$\begin{array}{c} f\left(l\right)=\left[\begin{array}{ccc} a_{1} & a_{2} & a_{3} \end{array}\right]\cdot {\left[\begin{array}{ccc} f_{1}\left(l\right) & f_{2}\left(l\right) & f_{3}\left(l\right) \end{array}\right]}^{T}. \end{array}$$

The optimal path of coal mine patrol robot can be obtained through optimization of the objective function f(l).

## 3. Path Planning of Hybrid Algorithm

As mentioned before, in the complex underground environment, the paths planned for coal mine patrol robot are often too long and unsmooth. To solve the problems, this section optimizes the above objective function with the improved hybrid AFSA-DWA path planning algorithm, based on the mathematical model for the path planning of coal mine patrol robot, and thereby obtains the optimal path.

### 3.1. Improved AFSA

**Figure 2:**
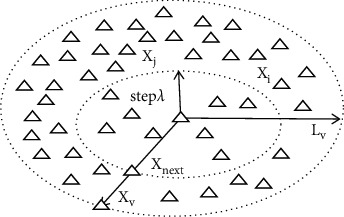
AFSA search process.

The AFSA was called for the global path planning of coal mine patrol robot. The three behaviors of AFSA, namely, foraging, clustering, and rear ending, were adopted to find the robot a safe, feasible path towards the designated location [15].  Figure 2 illustrates the AFSA search for coal mine patrol robot, where Xi, Xj, and Xv are the positions of artificial fishes *i*, *j*, and *v*, respectively, step*λ* is the step size of the motion, and Lv is the visual range.

The visual range Lv, motion step size step*λ*, and the fish swarm concentration Λ corresponding to the AFSA search can be expressed as

(11) $$\begin{array}{c} \left\{\begin{array}{c} L_{ij}=\left|X_{j}-X_{i}\right|<L_{v},\\ X_{next}=X_{i}+\text{Rand}\left(0,1\right)\times \text{step}\frac{X^{*}-X_{i}}{\left\|X^{*}-X_{i}\right\|},\\ \frac{n_{i}}{N}<\Lambda . \end{array}\right. \end{array}$$

L_ij_ is the distance between artificial fishes *i* and *j*; ni is the number of fishes in the current visual range; *N* is the total number of fishes in the swarm. If LV is far greater than L_ij_, it takes a long time for coal mine patrol robot to plan a path. If Λ is relatively large, the robot may not find the global optimal path easily. If step*λ* is relatively large, the path planned by the robot would contain many unsmooth turning points, and many redundant points.

To overcome the defects of traditional AFSA in path planning for coal mine patrol robot, the parameters of AFSA were improved as follows:

(1) To shorten the path planning time of coal mine patrol robot, the visual range of artificial fishes was improved to adaptively adjust the visual range LV^*∗*^:
  (12) $$\begin{array}{c} L_{v}^{*}=L_{v\max}-\frac{I_{i}}{I}\left(L_{v\max}-L_{v\min}\right), \end{array}$$
where L_vmin_ and L_vmax_ are the minimum and maximum visual ranges of artificial fishes, respectively, and I_i_ and I are the current number of iterations and maximum number of iterations, respectively.
(2) To reduce the turning points and redundant points on the path planned for coal mine patrol robot, and to make the path smoother, the motion step size of artificial fishes was improved, such that coal mine patrol robot can adaptively adjust the motion step size step^*∗*^*λ* between different nodes:
  (13) $$\begin{array}{c} {\text{step}}^{*}\lambda =\text{step}\lambda_{\text{max}}-\text{exp}\left(\frac{I_{i}}{I}\right)\left(\text{step}\lambda_{\text{max}}-\text{step}\lambda_{\text{min}}\right), \end{array}$$
where step*λ*_min_ and step*λ*_max_ are the minimum and maximum step sizes, respectively.

In order to solve the problem of local optimality in the optimization process of path planning of the coal mine inspection robot, by improving the fish concentration in the visual field of the artificial fish, the coal mine inspection robot can adaptively adjust the fish concentration of the transition point between the transition node and target point Λ^*∗*^:

(14) $$\begin{array}{c} \Lambda^{*}={\left(\frac{\Lambda_{\min}}{\Lambda_{\max}}\right)}^{\exp\left(1-I_{i}/I\right)}. \end{array}$$

In the formula, Λmin and Λmax are the minimum and maximum artificial fish concentrations in the field of vision of the artificial fish, respectively.

Although the above improvement of AFSA can optimize the path planning of coal mine inspection robot, when the target points of coal mine inspection robot remain unchanged, the target point density of the optimal neighborhood coal mine inspection robot is reduced, slowing down the convergence speed and increasing the path planning time. To solve the above problems, the genetic algorithm introduces artificial fish group algorithm. After introducing the genetic algorithm, the Metropolis criterion is used as the state update criterion of the artificial fish, some of the artificial fish with poor state are retained, the search space of the algorithm is expanded, the threshold value of the fitness function is set, the artificial fish larger than the threshold value is discarded, the remaining artificial fish are crossed by the genetic algorithm to produce new artificial fish for supplementation, and the mutation operation is performed on the artificial fish with the optimal state afterwards. The optimal artificial fish state is obtained, and adaptive probability factors are designed for crossover and mutation operations. The probability factor is large in the early stage of the algorithm search to ensure the diversity of artificial fish samples, and the global search ability of the algorithm is improved, and the probability factor becomes smaller in the late stage of the algorithm to accelerate the convergence speed of the algorithm as follows.

Firstly, the control parameter *T* is introduced to judge whether the position of fish *i* is updated by the Metropolis criterion. Whether the coal mine patrol robot updates the next set of target points can be judged by the following rule:

(15) $$\begin{array}{c} \Delta X_{i}=X_{j}-X_{i},\\ P=\left\{\begin{array}{c} 1,\\ \exp\left(\frac{-\Delta X_{i}}{T}\right)>\text{rand}\left(0,1\right), \end{array}\right.\begin{array}{c} \Delta X_{i}<0,\\ \Delta X_{i}>0. \end{array} \end{array}$$

rand(0,1) is a random number generated in the random distribution [0, 1] and *T* is the control parameter.

The optimal target point solved for coal mine patrol robot is updated, and the bulletin information is updated likewise. Then, the set of target points for the robot is sorted by the fitness function. The threshold K of the fitness function can be calculated by

(16) $$\begin{array}{c} \kappa =\frac{F_{m}}{F_{\text{best}}}, \end{array}$$

where Fm is the median of the fitness function.

The fishes with a fitness smaller than *K* are retained and subjected to adaptive crossover to generate new target points. The number of new target points is equal to the number of points in the set of target points. Then, the new position X_new_ of each target point of coal mine patrol robot can be obtained as

(17) $$\begin{array}{c} X_{\text{new}}=p_{c}X_{i}+\left(1-p_{c}\right)X_{j},\\ p_{c}=\left\{\begin{array}{cc} p_{c\min}\cos\left[\left(\frac{I_{i}}{I}\right)\times \left(\frac{\pi }{2}\right)\right], & I_{i}>\frac{I}{2},\\ p_{c\max}, & I_{i}<\frac{I}{2}. \end{array}\right. \end{array}$$

p_c_, p_cmin_, and p_cmax_ are the adaptive crossover probability, the minimum crossover probability, and the maximum crossover probability, respectively.

The optimal fish state after the crossover is subjected to mutation, producing the new optimal target position *X*^*∗*^best for coal mine patrol robot:

(18) $$\begin{array}{c} X_{\text{best}}^{*}=p_{m}X_{\text{best}},\\ p_{m}=\left\{\begin{array}{cc} p_{m\min}\cos\left[\left(\frac{I_{i}}{I}\right)\times \left(\frac{\pi }{2}\right)\right], & I_{i}>\frac{I}{2},\\ p_{m\max}, & I_{i}<\frac{I}{2}. \end{array}\right. \end{array}$$

p_m_, p_mmin_, and p_mmax_ are the adaptive mutation probability, the minimum mutation probability, and the maximum mutation probability, respectively.

After that, it is necessary to judge whether the fitness *X*^*∗*^best is greater than X_best_. If yes, the bulletin will be updated; otherwise, the iterations will continue until the global optimal path is found (i.e., the termination condition is satisfied).

**Figure 3:**
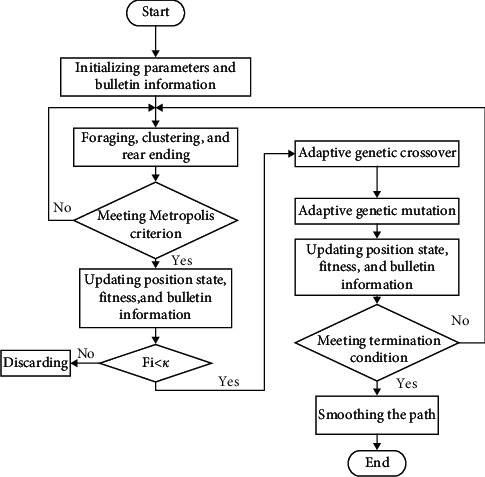
Flow of improved AFSA.

Figure 3 shows the flow of the improved AFSA.

The improved AFSA was applied to coal mine patrol robot. Specifically, the Metropolis criterion was introduced to guide the state update of artificial fishes. Then, a threshold was set up for the fitness function. The target points of coal mine patrol robot with a fitness smaller than threshold *k* were subjected to crossover, and mutation by the GA. In this way, the optimal target point X^*∗*^best was identified for the robot. In addition, adaptive probabilities were designed for the crossover and mutation operations. This ensures the diversity of the set of target points, enhances the global search ability of the algorithm, and accelerates the algorithm convergence.

On MATLAB, a 20 × 20 grid map was developed as the simulation environment. The path planning was simulated with the goal of minimizing the value of the objective function:

(19) $$\begin{array}{c} \min f\left(L\right)=a_{1}\cdot f_{1}\left(L\right)+a_{2}\cdot f_{2}\left(L\right)+a_{3}\cdot f_{3}\left(L\right). \end{array}$$

**Figure 4:**
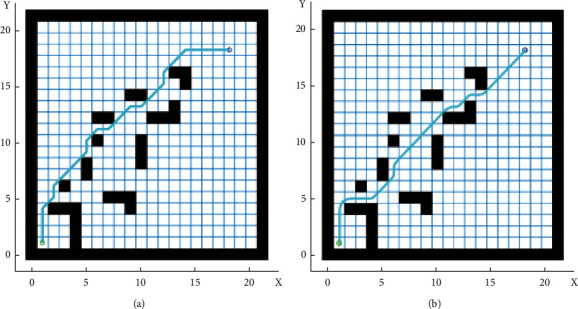
Path planned by original and improved AFSA. (a). Original AFSA. (b) Improved AFSA.

Figure 4 and Table 1 compare the simulation results of the original and improved AFSAs in the complex environment.

As shown in Figure 4 and Table 1, the optimal path obtained by the AFSA contained many redundant points and turning points, which hinder the motion of the patrol robot. The improved AFSA achieved a high search efficiency and planned a relatively smooth path. Compared with the path planned by the original AFSA, the path planned by the improved AFSA contains 3 fewer turning points and saves 5.3 m in length. The latter path is obviously more suitable for robot motions.

### 3.2. Improved Hybrid AFSA-DWA Path Planning Algorithm

In the complex underground environment of coal mine, it is crucial for the patrol robot to effectively avoid dynamic and temporary obstacles [16]. The above improved AFSA can search the optimal path globally. But Figure 4 shows that the improved AFSA led to many small turning angles and points in local areas during obstacle avoidance, which suppress the obstacle avoidance of coal mine patrol robot. To solve the limitation, the improved DWA was added to the local path planning of the robot to derive a new trajectory evaluation function. Besides, a hierarchical evaluation algorithm was proposed with the aid of reference lines. The new adaptive trajectory evaluation function can be expressed as

(20) $$\begin{array}{c} {ℜ}^{*}\left(v,\omega \right)=K\left(B\cdot d_{\min}\left(v,\omega \right)+C\cdot v\left(v,\omega \right)+\right.\\ \left.D\cdot d^{*}\left(v,\omega \right)+E\cdot d_{G}\left(v,\omega \right)+F\cdot \chi \right), \end{array}$$

where *d*^*∗*^(·) is the shortest distance from the end point of the velocity trajectory to the global optimal path, dG(·) is the shortest distance from the end point of the velocity trajectory to the local target point, and *χ* is an adaptive distance variable.

Firstly, the velocity samples with obstacles on the simulated trajectory were discarded. To avoid blind search, and to enhance algorithm efficiency, the information about the global planned path was introduced to the trajectory evaluation function.

The *d*^*∗*^(*v*, *ω*) can be expressed as

(21) $$\begin{array}{c} d_{G}\left(v,\omega \right)=\sqrt{{\left(x_{\text{end}}-x_{G}\right)}^{2}+{\left(y_{\text{end}}-y_{G}\right)}^{2}}, \end{array}$$

where (x_end_, y_end_) are the coordinates of the end point of the trajectory, (*x*^*∗*^, *y*^*∗*^) are the coordinates of the global optimal path, and (*xG*, *yG*) are the coordinates of the local target point of the patrol robot. The adaptive distance variable *χ* can be defined as

(22) $$\begin{array}{c} \chi =\frac{\Delta d_{t}}{\sqrt{{\left(x_{t}-x_{G}\right)}^{2}+{\left(y_{t}-y_{G}\right)}^{2}}}, \end{array}$$

where Δd_*t*_ is the trajectory moving distance in the dynamic window Δ*t* of the patrol robot and (*xt*, *yt*) are the coordinates of the patrol robot at time *t*.

**Figure 5:**
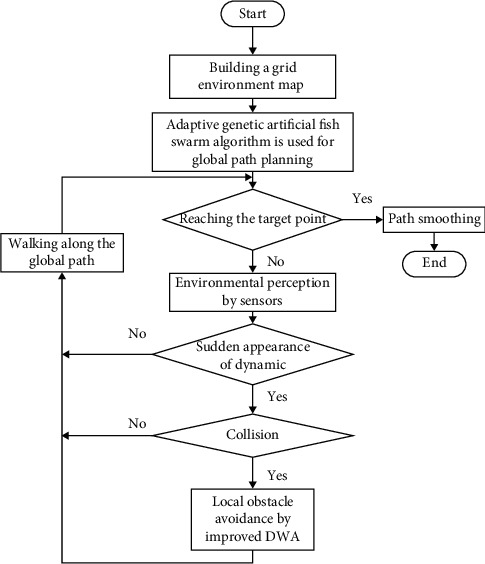
Flow of improved hybrid path planning algorithm.

Next, AFSA was fused with DWA. On the upper layer, the AFSA was called to obtain the global path information of coal mine patrol robot underground. Based on the global path information, the improved DWA was employed to perform local optimization, reducing the small angle turning points and redundant points in the global path information. The improved hybrid path planning algorithm makes an overall design of the path for the patrol robot, such that the robot can plan underground paths more robustly.  Figure 5 shows the overall design of the hybrid path planning algorithm.

The improved hybrid AFSA-DWA path planning algorithm improves the Lv, Λ, and step*λ* of AFSA for global path planning. Then, the GA is introduced to keep the size of the set of target points constant. After obtaining the global path information, the improved DWA is adopted to design a new trajectory evaluation function, with the global path as the reference. The new function makes the local path planning more accurate. In this way, the hybrid algorithm can detect the optimal path for coal mine patrol robot.

**Figure 6:**
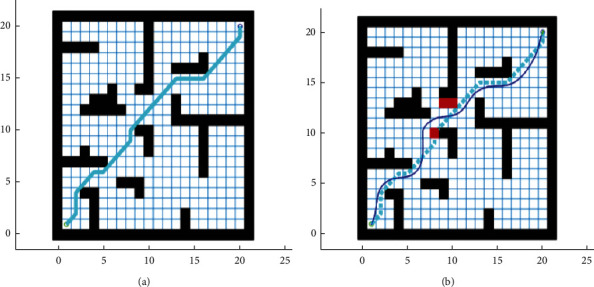
Path planned by two algorithms. (a) Static environment. (b) Dynamic obstacle avoidance.

After that, MATLAB simulation was carried out on a 20 × 20 grid map. Two scenarios were considered in the simulation: the presence of static obstacles, and the presence of dynamic obstacles. Formula (19) was adopted as the objective function.  Figure 6 and Table 2 show the simulation results of the original AFSA and our hybrid algorithm.

As shown in Figure 6 and Table 2, the improved hybrid AFSA-DWA algorithm shortened the optimal path length by 5.5 m from that of IAFSA and reduced the number of turning points on the path by five. The path planned by the hybrid algorithm is more suitable for robot motions. When the environment contained dynamic obstacles, the hybrid algorithm could avoid all the obstacles and obtain the shortest, most smooth path under the guidance of the global optimal path, wrapping up the task of path planning.

## 4. Simulations

### 4.1. Simulation Plan

Mine safety inspection is one of the key tasks to ensure the safety of mining. At present, the manual inspection method has problems such as large workload, low efficiency, excessive reliance on the experience of inspectors, and waste of human resources. Mine inspection robot is of great significance to replace manual inspection, achieve intuitive visual management, reduce labor intensity, improve the level of coal mine management, promote the mine work management mode to digital, modern direction, and ensure mine safety production.

**Figure 7:**
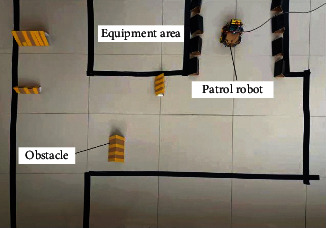
Simulation environment.

The underground workface of coal mine patrol robot has a complex environment and multiple obstacles. Multiple way points and mobile obstacles were set up to simulate the underground environment of coal mine.  Figure 7 shows the simulation environment.

**Figure 8:**
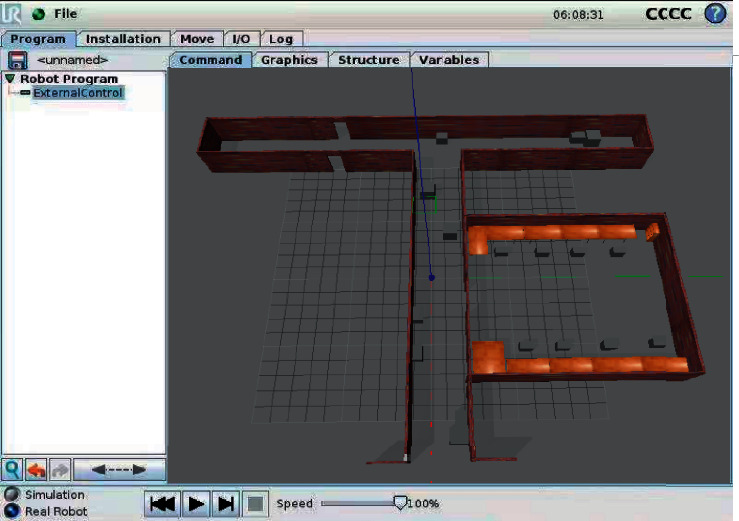
Simulation platform for robot control.

The simulation environment was constructed in the robot operating system (ROS), producing a simulation platform for the control of coal mine patrol robot (Figure 8). In the ROS-based underground simulation environment, the coal mine patrol robot was connected to the simulation platform for robot control via USB. Then, the improved hybrid AFSA-DWA path planning algorithm was imported, and random target points were selected in the established environment via the platform. The autonomous motions and obstacle avoidance of coal mine patrol robot were observed to obtain the global optimal path.

### 4.2. Results Analysis

**Figure 9:**
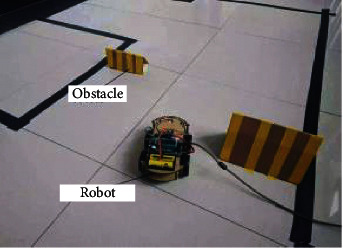
Motion and obstacle avoidance of the robot.

**Figure 10:**
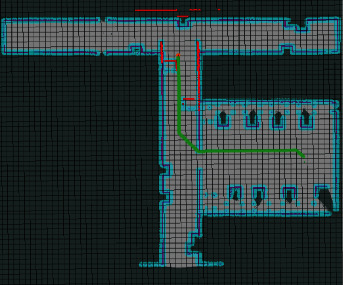
Path planned by the original AFSA.

**Figure 11:**
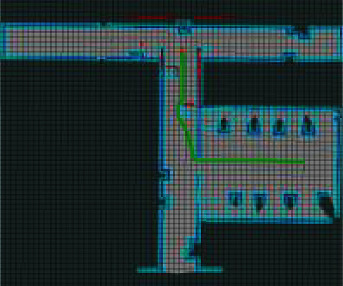
Path planned by the improved hybrid IAFSA-DWA algorithm.

To verify the superiority of the improved hybrid AFSA-DWA algorithm in path planning of coal mine patrol robot, mobile obstacles were added to the established environment, and the original AFSA and the improved hybrid AFSA-DWA algorithm were tested on the simulation platform.  Figure 9 shows the motion and obstacle avoidance of the robot.  Table 3 presents the theoretical and practical paths planned by the two algorithms. Figures 10 and 11 show the path planned by the original AFSA and the improved hybrid AFSA-DWA algorithm, respectively.

As shown in Table 3, the improved hybrid path planning algorithm performed well in planning a smooth path with relatively few redundant points and turning points for coal mine patrol robot. Compared with the AFSA, the improved hybrid AFSA-DWA algorithm planned local path very efficiently, shortened the path by 0.12 m, and saved 3.14 s of planning time.

As shown in Figures 10 and 11, the path planned by the proposed improved hybrid IAFSA-DWA algorithm was smoother, involving much fewer turning points and redundant points, than that planned by the traditional AFSA. The proposed algorithm provides an effective solution to the nonsmoothness and heavy presence of turning points and redundant points faced by path planning of coal mine patrol robot. The simulation results confirm that the improved hybrid AFSA-DWA algorithm is a superior, feasible, and highly suitable method for path planning of robots.

## 5. Conclusions

Mine safety inspection is one of the key tasks to ensure the safety of mining. Currently, the manual inspection method has problems such as high workload, low efficiency, excessive reliance on inspector experience, and wasted human resources. Mine inspection robot is of great significance to replace the manual inspection, achieve intuitive visual management, reduce labor intensity, improve the level of coal mine management, promote the mine work management mode to digital, modern direction, and to ensure the safety of mine production.

To solve the problems of path planning for coal mine patrol robot (e.g., excessive length and nonsmoothness of the planned path), the merits of AFSA and DWA were integrated to propose an improved hybrid path planning algorithm. MATLAB simulations confirm that the proposed algorithm can significantly reduce the number of redundant points and turning points on the planned path and complete path planning more effectively than the original AFSA. In addition, a simulation platform for the control of coal mine patrol robot was established on coal mine patrol robot and ROS development platform. Through the simulations on the developed platform, it was observed that the improved hybrid path planning algorithm reduced the length of the planned path by 0.12 m, saved 3.14 s of planning time, and greatly lowered the number of turning points and redundant points, as compared to the original AFSA. Therefore, the proposed improved hybrid path planning algorithm is a feasible and superior way to overcome the excessive length and nonsmoothness of the path planned for coal mine patrol robot.

At present, the complex environment of underground coal mine has more influence on the path planning of coal mine inspection robots, and the large dust and strong noise environment of underground coal mine inspection robots restrict the collection of basic data such as equipment and operating space environment by the sensors of coal mine inspection robots, and the subsequent research needs to realize the all-round monitoring of working environment and equipment and further continue to improve the performance of path planning algorithm through the linkage of inspection robots and technicians to feed each other, so as to get a smoother and safer path.

## Figures and Tables

**Table 1:** Path planning results of original and improved AFSAs.

Environment	Parameter	AFSA	Improved AFSA
Complex environment	Number of turning points	12	9
	Path length	29.556	24.384

**Table 2:** Path planning parameters of two algorithms.

Environment	Parameter	IAFSA	Hybrid algorithm
Static environment	Number of turning points	11	6
	Path length	29.213	24.967

**Table 3:** Theoretical and practical paths.

Path name	Path length (m)	Length increment (m)	Time cost (s)
Theoretical path	2.2		29.75
Original planned path	2.48	0.28	36.48
Improved planned path	2.32	0.16	32.34

## Data Availability

The data used to support the findings of this study are available from the corresponding author upon request.
